# Reliability of fNIRS for noninvasive monitoring of brain function and emotion in sheep

**DOI:** 10.1038/s41598-020-71704-5

**Published:** 2020-09-07

**Authors:** Matteo Chincarini, Emanuela Dalla Costa, Lina Qiu, Lorenzo Spinelli, Simona Cannas, Clara Palestrini, Elisabetta Canali, Michela Minero, Bruno Cozzi, Nicola Ferri, Daniele Ancora, Francesco De Pasquale, Giorgio Vignola, Alessandro Torricelli

**Affiliations:** 1 Facoltà Di Medicina Veterinaria, Università Degli Studi Di Teramo, Teramo, Italy; 2 Dipartimento Di Medicina Veterinaria, Università Degli Studi Di Milano, Milan, Italy; 3 Dipartimento Di Fisica, Politecnico Di Milano, Milan, Italy; 4 School of Software, South China Normal University, Guangzhou, China; 5 Istituto Di Fotonica E Nanotecnologie, Consiglio Nazionale Delle Ricerche, Milan, Italy; 6 Dipartimento Di Biomedicina Comparata E Alimentazione, Università Degli Studi Di Padova, Padova, Italy; 7 Istituto Zooprofilattico Sperimentale Dell’Abruzzo E del Molise G. Caporale, Teramo, Italy

**Keywords:** Optical spectroscopy, Animal behaviour, Imaging and sensing, Optical spectroscopy

## Abstract

The aim of this work was to critically assess if functional near infrared spectroscopy (fNIRS) can be profitably used as a tool for noninvasive recording of brain functions and emotions in sheep. We considered an experimental design including advances in instrumentation (customized wireless multi-distance fNIRS system), more accurate physical modelling (two-layer model for photon diffusion and 3D Monte Carlo simulations), support from neuroanatomical tools (positioning of the fNIRS probe by MRI and DTI data of the very same animals), and rigorous protocols (motor task, startling test) for testing the behavioral response of freely moving sheep. Almost no hemodynamic response was found in the extra-cerebral region in both the motor task and the startling test. In the motor task, as expected we found a canonical hemodynamic response in the cerebral region when sheep were walking. In the startling test, the measured hemodynamic response in the cerebral region was mainly from movement. Overall, these results indicate that with the current setup and probe positioning we are primarily measuring the motor area of the sheep brain, and not probing the too deeply located cortical areas related to processing of emotions.

## Introduction

How animals are treated matters both to animals and to people. The focus on animal welfare stems from the recognition that animals are sentient beings and finding noninvasive indicators of animal emotion and cognition processes is an important goal in disciplines ranging from neuroscience to animal welfare. However, the neurophysiological basis of behavior of domestic herbivores, raised for meat, milk or wool production, are still largely unknown. The sheep may be adapted as a valid model for its ability to perform a variety of complex tasks and behaviors involving goal-oriented motor coordination, emotion, facial recognition and memory-based performance^1–4^. In addition, the convoluted brain of the sheep may be considered as an alternative to rodents in translational experimental neuroscience^1,5^. As cognitive processes or emotional states can often be reflected in brain responses^6^, a better understanding of affective states of sheep in various environments or as a response to different stimuli will benefit both neuroscience and animal welfare.

Understanding how cognitive processes and emotional states are expressed in animal brain is still a challenge, especially in a noninvasive manner. Previously, several methods successfully used in human brain mapping have been employed in functional imaging of the animal brain, including functional magnetic resonance imaging (fMRI)^7–14^, electroencephalography and sensory evoked potentials^15–19^. While in human studies tasks and stimuli can be presented to an awake and collaborating subject, most measurement techniques require the animal to be under anesthesia. Therefore, its brain response can be strongly affected.

Functional near-infrared spectroscopy (fNIRS) is a novel technique that employs near-infrared light to non-invasively measure the concentration of oxygenated hemoglobin [O_2_Hb] and deoxygenated hemoglobin [HHb] in cerebral human tissue, or typically their changes with respect to a baseline period, [ΔO_2_Hb] and [ΔHHb]. Like fMRI, fNIRS relies on the neurovascular coupling mechanism^20^, and on the ability of near-infrared light to penetrate deeply in biological tissue^21^. Being non-invasive, safe, portable, and characterized by a low susceptibility to motion artifacts, fNIRS has been widely and successfully employed in human studies^22^. The most commonly utilized fNIRS approach is the continuous wave fNIRS (CW-fNIRS) employing steady state light sources and detectors^21^. Since CW-fNIRS systems can be wireless and miniaturized, several applications have also been carried out in free-moving domestic animals, e.g. goats^23^, dogs^24^, and sheep^25–29^. In these studies, fNIRS monitoring has been considered and used as an additional technique to measure emotional and cognitive responses in animals exposed to different stimuli or different environments. Noticeably, results were sometimes inconsistent among studies^6,30^, suggesting that measurement accuracy and reliability need to be improved.

As a matter of fact, all previous studies on farm animals^23–29^ employed a fNIRS system that was designed for human neonatology and not optimized for species-specific animal anatomy^31^. In particular, they all made use of a fixed configuration of source detector pairs with short (15 mm) and long (25 mm) acquisition channels that are sub-optimal for sampling cortical region and for discriminating extra-cerebral regions (i.e. scalp, skull and CSF) in animals like sheep with an average depth from scalp to brain cortex that can be up to 10 mm^32^, much closer to adult than to neonate humans.

Further, differently from what it is normally done in fNIRS studies on humans where probe placement can be guided by proper neuro-anatomical images and functional atlas^33–35^, the placement of the probe on the animal head was not aided by magnetic resonance imaging (MRI) data and rarely it was not consistently supported by neuro-anatomical or functional atlas of the animal brain^25^. Consequently, fNIRS signals were attributed to the frontal or pre-frontal region, also hypothesizing a vascular stealing mechanism in the brain cortex^23,26–28^, but without determining the sensitivity of the technique to that specific cortical region.

Moreover, the methods employed for fNIRS data analysis potentially suffered from lack of accuracy. In fact, the more superficial hemodynamic events occurring in the scalp could interfere with the hemodynamic changes from the cortex unless a proper geometry method to model the head of the animal is adopted.

Finally, fNIRS data processing made use of a priori information on the differential pathlength factor (DPF) taken from the literature and obtained from a single dead animal^36^. To improve accuracy of fNIRS data analysis, the DPF is in fact used to derive hemoglobin changes from the measured intensity changes by means of the Modified Beer Lambert law or similar model based approaches^21^. Wrong DPF data can yield to inaccurate estimates of hemoglobin concentration.

From the above observations, it becomes evident that the fNIRS technique is still an innovative, not fully validated approach for studying cortical activity in domestic animals. The aim of this work was to improve reliability and accuracy of fNIRS measurements in sheep and critically assess whether fNIRS can be profitably used as a tool for noninvasive recording of brain functions and emotions in this species. To this purpose we have considered an experimental design that encompasses advances in instrumentation (customized wireless multi-distance fNIRS system), more accurate physical modelling (two-layer model for photon diffusion and 3D Monte Carlo simulations), support from neuroanatomical tools (positioning of the fNIRS probe by MRI and DTI data of the very same animals), and rigorous protocols (motor task, startling test) for testing the behavioral response of freely moving sheep.

## Results

### Hemodynamic changes during the motor task

**Figure 1:**
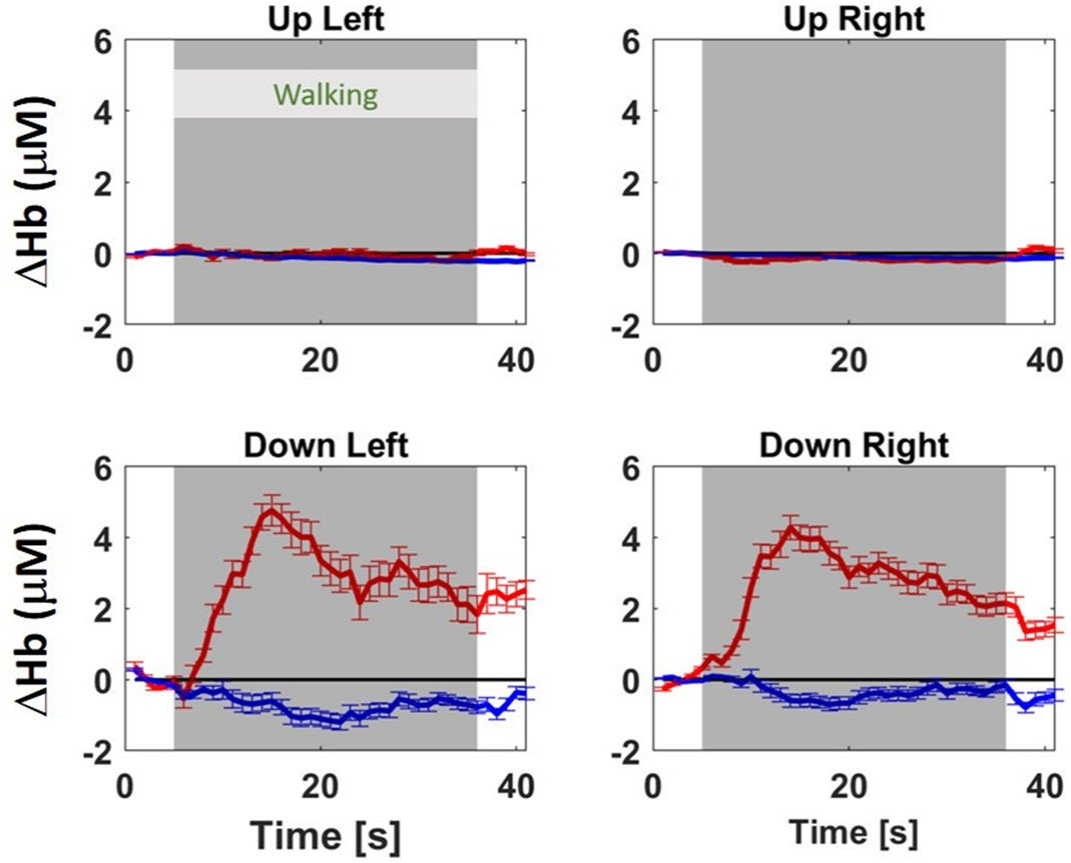
Group-average of [ΔO_2_Hb] (red lines) and [ΔHHb] (blue lines), in μM, and their standard deviation during the motor task for extra-cerebral (up) tissue (top row) and cerebral (down) tissue (bottom row) and for the left (left column) and right (right column) hemisphere. In every plot, the first 5 s are the baseline (sheep stand still), followed by 30 s walking (marked as gray area), and finally 5 s recovery period (sheep stand still). The horizontal black line in every sub-figure indicates the zero value.

Out of the 13 sheep that performed the motor task, two sheep were excluded from the analysis because of frequent head shaking, as shown by the evaluation of the video recordings (for details on the ethogram see Supplementary Table ST2). In the remaining 11 sheep we calculated the group-average [ΔO_2_Hb] and [ΔHHb] of the left and right hemispheres (Fig. 1, bottom row). The group-average in the two hemispheres shows a canonical hemodynamic response with an increase of [ΔO_2_Hb] and a non symmetrical decrease in [ΔHHb] in the cerebral tissue during the movement (p < 0.005). Almost no hemodynamic changes were observed in the extra-cerebral tissue (Fig. 1 top row).

### Hemodynamic changes during the starling test

**Figure 2:**
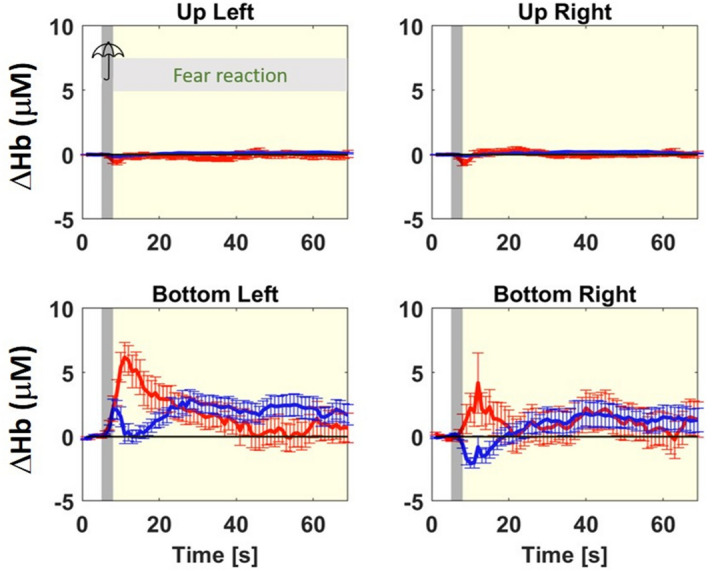
Group-average of [ΔO_2_Hb] (red lines) and [ΔHHb] (blue lines) , in μM, and their standard deviation during the startling test for extra-cerebral (up) tissue (top row) and cerebral (down) tissue (bottom row) and for the left (left column) and right (right column) hemisphere. In every subplot, the first 5 s is the baseline, followed by a 3 s startle stimulus (marked as gray area), and finally a 60 s fear reaction. The horizontal black line in every subplot indicates the zero value.

Thirteen sheep underwent to the startling test, three of them were excluded due to their behavior during the task: two sheep showed frequent head shaking; one showed no no flight response or freezing after the umbrella opening. In the remaining 10 sheep we also excluded blocks if the animals were chewing or shaking the head. We thus retained approximately 80% of the recorded blocks (39 in total), that were then averaged to obtain the group-average [ΔO_2_Hb] and [ΔHHb]. In the extra-cerebral tissue there is almost no hemodynamic change throughout the test, as shown in Fig. 2, top row. In both hemispheres, the cerebral tissue showed a transient increase in [O_2_Hb] after the startle stimulus (as shown in Fig. 2, bottom row). Further, the right hemisphere also shows a transient classical brain activation pattern with increase in [O_2_Hb] and decrease in [HHb].

Analysis of animal behavior revealed two distinct reactions to the startle stimulus: a flight response was observed in 28 blocks from 10 sheep, while freezing was observed in 11 blocks from 8 sheep. Therefore, we calculated the group-average [ΔO_2_Hb] and [ΔHHb] for the two groups, labelled Move group and Stand group.

**Figure 3:**
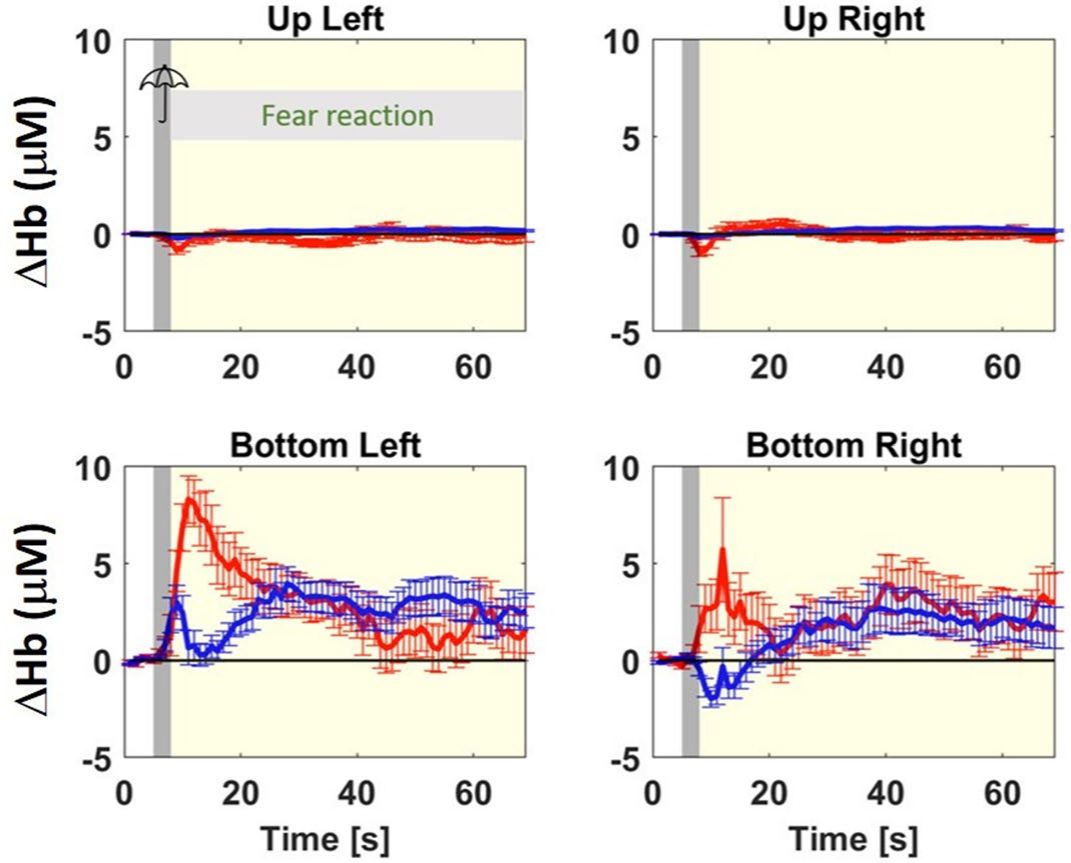
Same as Fig. 2 but group-average of the move group (sheep were moving during and after the 3 s startling stimulus).

**Figure 4:**
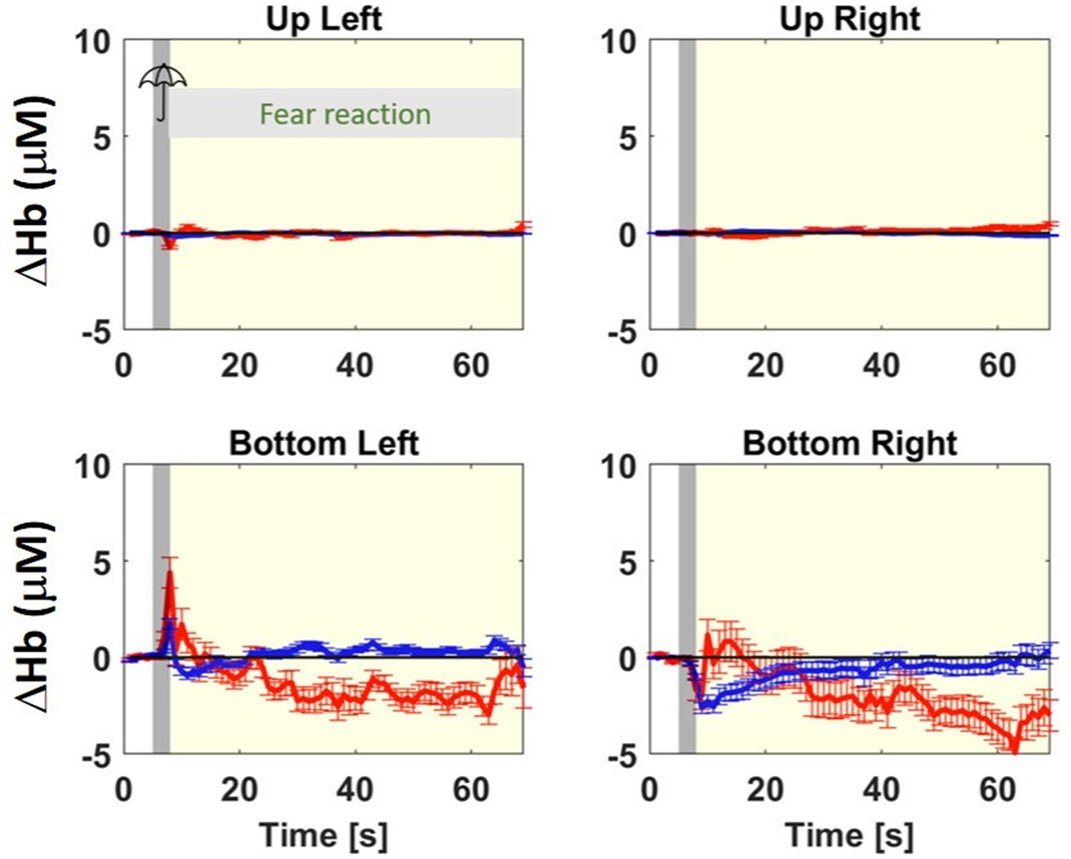
Same as Fig. 2 but group-average of the stand group (sheep were still during and after the 3 s startling stimulus).

Both groups showed almost no hemodynamic response in the extra-cerebral tissue (Fig. 3 top row, and Fig. 4, top row). In the cerebral tissue, for the Move group (Fig. 3 bottom row) we have a canonical hemodynamic response (i.e. increase in [O_2_Hb] and decrease of [HHb]) in both left and right hemisphere. We also notice an increase in both oxygenated and deoxygenated hemoglobin in the left hemisphere cerebral tissue before the canonical response, during the opening of the umbrella, (see the gray area in Fig. 3 bottom row).

Conversely, for the Stand group the pattern of hemodynamic changes is less clear and different in the two hemispheres. In the left hemisphere we see a rapid increase of [O_2_Hb] and [HHb] followed by a slow decay of [O_2_Hb] to values smaller than the baseline, while for [HHb] we observe a return to baseline values with a small undershoot (see Fig. 4, bottom left panel). In the right hemisphere after an initial decrease for both [O_2_Hb] and [HHb], we notice a return to the baseline for [HHb] and irregular oscillations for [O_2_Hb] that lead to values lower than the baseline (see Fig. 4, bottom right panel).

## Discussion

This study investigated the cerebral activity of freely moving sheep by using a wearable CW-fNIRS system applied to the animals performing a motor task and a startling test. Here we describe an experimental design that encompasses advances in instrumentation, more accurate physical modelling and rigorous protocols for assessing the animal behavioral response, with the aim of improving the fNIRS methodology applied to domestic animals.

Firstly, we employed a customized multi-distance wireless CW-fNIRS system with freely adaptable placement of sources and detectors. The short and long source-detector distances used were ρ = 10 mm and ρ = 30 mm, respectively. This configuration, notwithstanding the limitations imposed by the size and anatomical features of the animals' heads, allowed both to enhance photon penetration depth in the sheep head thanks to a long distance, longer than in previous studies, and to better discriminate extra- and intra-cerebral contributions, thanks to a reduced short distance. A study on the photon penetration depth at ρ = 10 mm and ρ = 30 mm in both simplified (homogeneous slab) and realistic (3D mesh from MRI data) geometry is reported in the Supplementary Section S3 to help the reader appreciate the different features of photon penetration at different distances.

We subsequently guided the positioning of the fNIRS probe by MRI data of the very same animals, since a neuro-anatomical and functional atlas for Sarda sheep (used in this study) was not available. We also checked the functional origin of the signal by diffusion tensor imaging (DTI) MRI on the animal brain. From MRI and DTI data we confirmed that the location of the fNIRS probe was over the motor area of the cortex (see Supplementary Section S1).

Moreover, we noninvasively measured the optical properties (absorption coefficient and reduced scattering coefficient) of the head of all animals by employing a state-of-the-art multi-wavelength time-resolved diffuse spectroscopy system (see Supplementary Section S2). Photon distributions of time-of-flight (DTOF) were acquired at several wavelengths and fitted to a model for photon diffusion. Data of the optical properties were then used to improve a model-based data analysis. From the DTOF we also calculated the DPF on living sheep, therefore providing brand new data for this parameter (see Supplementary Table ST1). To the best of our current knowledge, the literature contains references only to post mortem DPF data obtained from a single animal^36^. Our DPF data can contribute to enhancing the accuracy of analysis^21^ of other fNIRS studies enrolling sheep, thanks to the high uniformity that is expected from animal to animal due to genetic selection and reproduction procedures in animal production science^37,38^.

Finally, to improve the estimate of the hemodynamic response in brain cortex, we have introduced, and validated by simulations (as reported in Supplementary Section S4), a model-based approach to data analysis of fNIRS signals from short and long distances. The model employs a two-layer geometry to better mimic the extra- and intra-cerebral tissue layers in the sheep’s head, aiming at advancing the previous approaches based on a homogeneous model.

All the previously described activities were functional to the design of an experimental test capable of noninvasively estimate the cerebral hemodynamics of freely moving sheep during behavioral tasks. The motor task and the startling test were chosen as election assessments because (*a*) we wanted a straightforward test (the motor task) as a validation of our methodological approach; and (*b*) the startling test could give us insights into the possibility to monitor emotion and cognitive responses in sheep.

Before fNIRS recordings, sheep underwent a training period to minimize stress possibly associated with the tasks performance and behavioral reactions that could hamper the quality of fNIRS data acquisition^39^. In particular, the sheep were habituated to be separated in small groups of animals of the same flock, to be handled by humans and to wear fake fNIRS devices (including cap, cables and chest straps). They were then trained to walk and stop in response to a vocal cue.

The results of the motor task were encouraging since a canonical hemodynamic response was observed, with increase in O_2_Hb and decrease in HHb in the cortex located immediately below the fNIRS probe and without appreciable contribution from non-nervous tissues, including skin, subdermal layers, bone and dura mater. On the basis of the results of fNIRS experiments on human subjects where a similar hemodynamic response was found for motor task and walking experiments^40,41^, we can conclude that the fNIRS technique with the proposed settings is able to monitor the cortical response associated to the execution of a motor task in sheep.

In the startling test, we found that—when moving during the startling stimulus (flight reaction)—the sheep showed a transient brain activation (i.e. increased hemodynamic response) that followed the actual stimulus. However, when the sheep was freezing, so remained standing still after the startle stimulus, the responses of both hemispheres were minimal, only showing a limited drop in [O_2_Hb]. Even though the number of blocks of sheep showing a freezing response was limited, these results thus suggest that the brain response observed after the startle stimulus is likely to come from the movement rather than the startle itself, as shown in Figs. 3 and 4.

These results benefit of the simultaneous study of extra-cerebral and cerebral tissue layers that helped to identify a classical neuroactivation when occurring.

The neuroimaging data on sheep obtained in this study (see Supplementary Section S1) indicated that in our experimental setting the fNIRS sensors were placed above the motor cortex of the sheep and recorded the vascular dynamics related to Brodmann area 4 (for extended discussion and references on the position and connections of the motor cortex of the sheep see Ref.^42^). Overall, the results of the startling test and of the motor task furtherly indicate that we are mainly measuring the motor area of the sheep brain, and that we are not probing the too deeply located “prefrontal” cortex of the supraorbital gyrus or other cortical areas related to processing of emotions. In fact, DTI MRI data also confirm that the fiber tracts that originate from the cortical area under the fNIRS probe belong to the pyramidal and extrapyramidal motor control pathways that project to the generators of motor schemes in the brainstem or to the spinal cord, either homolaterally or (mostly) contralaterally^42^).

The positioning of the fNIRS probe, constrained by the dimensions of the animals’ heads, and the basic characteristics of the fNIRS device (e.g. wavelength range, optical power) were similar to previous published work^23,26–28^. Differently from other studies, thanks to the use of proper short distance and long distance channels, we were able to clearly discriminate the contributions from extra-cerebral and cerebral tissue. Our experience suggests that inconsistent and conflicting fNIRS results on the emotional and cognitive responses of animals reported in literature^6,30^ can be explained by the fact that it is very unlikely that the photons may reach the sheep equivalent of the human pre-frontal cortex and other areas dedicated to processing of cognitive stimuli, placed too distant from scalp, and the respiratory sinuses immediately below it in the animal calvaria (see Supplementary Section S1 and Supplementary Section S3).

Although we carefully evaluated all the potential confounding factors of previous experimental works, we are also aware that there are still some limitations that could affect the outcome of this research.

The choice of the location of the fNIRS probe was determined by the anatomical configuration of the head of the sheep. The natural curvature of the skull allowed positioning the sensors only on the top part of the head and prevented the use of larger source detector distances (ρ > 30 mm). Further, a shorter source detector distance (ρ < 10 mm) could be beneficial to better discriminating extra-cerebral and intra cerebral layers, but it was prevented in our work by the specific configuration of the fNIRS sensors.

We only had a limited number of fNIRS sensors that prevented mapping the hemodynamic response with a better lateral resolution, e.g. differentiating anterior and posterior parts of cerebral cortex. Mapping is important if one is interested in understanding the specific mechanism and *all* the different cortical areas involved in the execution of the movement. Movement is in fact a complex task involving multiple regions of the brain^43^; for a review of the organization of the motor cortex in large herbivores and extended references see Ref.^44^. Therefore, in our work when the sheep were moving, the specific working mechanism in the brain and the response of multiple brain areas could not be studied in detail and need to be further investigated.

We have measured the baseline optical properties of the head of the sheep by using a homogeneous model. The time-resolved diffuse spectroscopy system that we used had a limited responsivity that prevented acquiring multi-distance measurements at several discrete wavelengths (needed for proper application of a two-layer model) within an overall acquisition time sufficiently short not to induce stress in the measured animal. Refining the estimate of baseline optical properties could further improve the quantification of the hemodynamic response.

Similarly, assuming that the reduced scattering coefficient is constant in time (during the experiments) and space (in extra- and intra-cerebral body layers) could affect the accuracy of the hemodynamic response. However, it is unlikely that the reduced scattering coefficient varies appreciably during functional tests.

We have disregarded the contribution of other chromophores (e.g. water, lipid, collage) in the estimate of the concentration of (baseline and transient) O_2_Hb and HHb. Like for the reduced scattering coefficient we do not expect these parameters to vary significantly during the functional tests.

In the pre-processing phase, the removal of blocks was based on the visual inspection of the video recordings. Another more objective method for artifacts removal, especially motion artifacts, is the use of accelerometers^25^. Since the used fNIRS device was not equipped with a built sensor for acceleration we have preferred not to complicate further the experimental setup by adding another sensor on the limited space on the head of the animal.

Finally, the current work does not give a definitive conclusion about the brain response pattern of sheep undergoing behavioral tasks. A clear and definite brain response pattern is actually difficult to obtain from one or few experiments, which require repeated trials or a larger animal sample. The fNIRS technique represents still an innovative approach for studying cortex activity in freely moving animals and the number of studies performed using fNIRS on animals is still low.

## Methods

### Ethical statement

All methods on animals were carried out in accordance with relevant guidelines and regulations, and all experimental protocols were approved by a named institutional and/or licensing committee. In particular, the study design, which was created in compliance with Italian legislation on animal experiments, was approved by the national ethical commission (Ministero della Salute, Direzione Generale della Sanità Animale e dei Farmaci Veterinari, Ufficio 6, authorization n◦457/2016-PR, 919/2017-PR). If any animal was deemed to be in greater than mild stress (assessed live by an independent veterinarian), then it would immediately be removed from the study.

### Animals, housing and husbandry

Thirteen 8-month-old Sarda sheep were selected from the same flock. All sheep were no gestating nor lactating and they had never been involved in any study before. They were group housed in a 45 m^2^ pen (resting box), fed with hay twice a day (8 a.m. and 6 p.m.). Diet was supplemented with a commercial concentrate (Mangimi Ariston Srl, Teramo, Italy; 250–300 g/sheep). All sheep had free access to water and straw was provided for bedding.

### Experimental area and behavioral tasks

**Figure 5:**
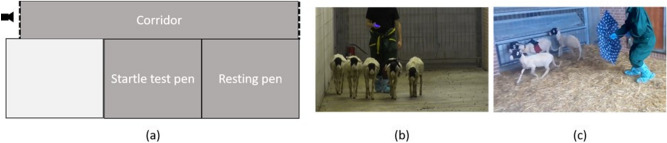
Schematic representation of the experimental area (**a**), the dotted lines represent mobile fences, the white line represents a wood door between the two pens; corridor (**b**) and startling test pen (**c**).

To measure the cerebral activity in different conditions, all sheep underwent to a motor task and a startling test. The experimental area (Fig. 5) was familiar to the sheep and it comprised a corridor (2 m wide × 20 m long) and a startle test pen. The end of the corridor was closed with metal panels routinely used for building animal enclosures (height 1.5 m). The startle test pen was similar to the pen were the sheep were housed. To avoid any negative impact of being isolated from the flock during behavioral tasks, sheep were always kept in small groups (3–5 subjects).

The motor task consisted in sheep walking at the same speed along the corridor and stop when asked. Therefore, five sheep at each time were moved in the corridor and trained to walk slowly and calmly for 30 s and stop for the following 30 s. For this reason, they were trained through classical conditioning to start and stop walking on a vocal cue. The training was performed every day, for 26 days. We gradually habituated the sheep to wear a fake fNIRS probe, including cap, cables and chest straps, following the scheme: days 1–7 sheep were walking without any probe; days 8–14 sheep were walking wearing the supporting masks for the probe; days 15–26 sheep were walking with wearing the supporting masks for the probe and a sponge mimicking the probe pressure. At the end of the training phase no sign of stress was shown by the sheep.

The startling test consisted in an umbrella suddenly opened near the animals, able to evoke a fear reaction. To reduce the impact of habituation to the startling stimulus, sheep selected for fNIRS measurement were never been exposed to this stimulus before.

### fNIRS data recording and data analysis

**Figure 6:**
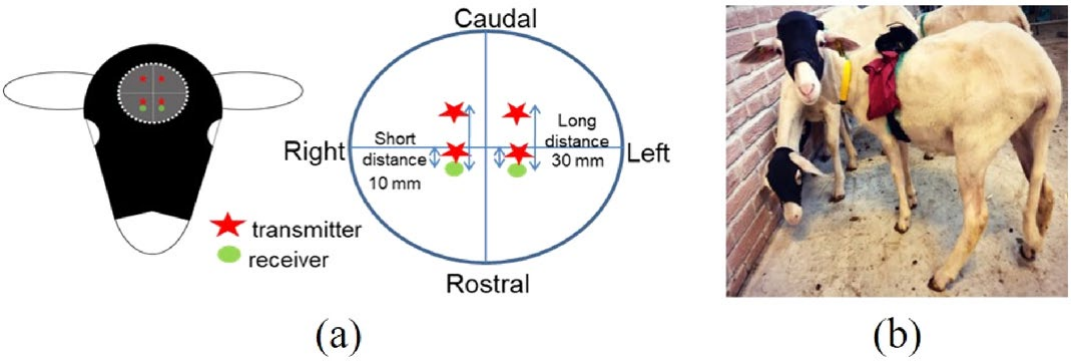
(**a**) The configuration of optical probes on the surface of sheep head: red stars represent the transmitters and green circles represent the receivers. (**b**) Sheep equipped with fNIRS devices.

To accurately measure concentration changes in oxygenated hemoglobin ([ΔO_2_Hb]) and deoxygenated hemoglobin ([ΔHHb]) in each sheep undergoing different behavioral tasks, a wearable CW-fNIRS system (OctaMon, Artinis Medical Systems, The Netherlands) operating at a sampling rate of 10 Hz, at two wavelengths (751 nm and 839 nm), and equipped with 4 transmitters (light sources) and 2 receivers (detectors) was used. The device was customized by the manufacturer to enable multi-distance acquisition. Two light sources and one detector were placed on each hemisphere at a source detector distance ρ = 10 mm and ρ = 30 mm, respectively, as shown in Fig. 6a. This configuration allowed recording of signals from both superficial tissues (e.g. the scalp and skull) and from the cortical tissue. During fNIRS recording, the sensors were applied on the shaved sheep head and held in place with a customized head cap as shown in Fig. 6b.

The fNIRS recordings were completed in two consecutive days (first day motor task, second day startling test) for all sheep. In the motor task, for each sheep, we recorded 10 repetitions (blocks) each with 30 s of walking followed by 30 s of rest (animal stand still). For the startling test, for each sheep, we recorded 5 blocks consisting of a 30 s baseline (sheep stand still), followed by the umbrella opening (3 s) and then 60 s of fear reaction. All the blocks of both motor task and startling test were filmed with a video-camera (Panasonic, HDC-SD99, Panasonic, Japan) and synchronized with fNIRS recordings. Video recordings were then analyzed using the software Solomon Coder (version beta 11.01. 22) to identify whether the sheep showed behaviors that could interfere with fNIRS recording such as head shaking, running and jumping. For the startle test, fear reaction (presence of flight and/or freezing response after the stimulus) was evaluated. The ethogram of behaviors considered is presented in the Supplementary Table ST2.

The fNIRS data were analyzed by a script in Matlab2015a (Matlab, The MathWorks Inc., Natick, Massachusetts). The raw fNIRS data extracted by the software Oxysoft (v3.0.95, Artinis Medical Systems, The Netherlands) were the optical density (OD) for each wavelength and each channel. For each task the last 5 s before each block were chosen as baseline. The change in OD relative to the baseline (i.e. ΔOD) were then calculated by subtracting the average OD of the baseline periods from all OD values.

According to anatomical and MRI measurements of sheep of the same age, the average distance between scalp and brain cortex was estimated to be *s* = 10 mm. Given this, we assumed (see Supplementary Section S3) that the ΔOD data from ρ = 10 mm (ΔOD_SHORT_) referred to photons that travelled exclusively in the extra-cerebral tissue (scalp, skull, and CSF). Therefore, using a Levenberg–Marquardt algorithm for nonlinear iterative least squares minimization (lsqcurvefit function in Matlab), and assuming the reduced scattering coefficient of the upper layer (representing extra-cerebral tissue) equal to the baseline value (μ_s_′^UP^ = μ_s0_′), we fitted ΔOD_SHORT_ data to a homogenous model^45^ to estimate the absorption changes relative to the baseline in the upper layer (Δμ_a_^UP^). The absolute value of the absorption coefficient in the upper layer was then calculated as μ_a_^UP^ = μ_a0_ + Δμ_a_^UP^, by adding to Δμ_a_^UP^ the baseline absorption coefficient μ_a0_ (see Supplementary Section S2 for description of μ_a0_ estimate).

Next we used ΔOD recorded at ρ = 30 mm (ΔOD_LONG_) and a two-layer model for photon migration^46^, to derive Δμ_a_^DOWN^, i.e. the change in the absorption coefficient in the bottom layer (representing cerebral tissue). In this step we assumed as a priori information the thickness of the upper layer (*s* = 10 mm), the absorption coefficient of the upper layer (μ_a_^UP^) and the reduced scattering coefficient of the upper and bottom layer (μ_s_′^UP^ = μ_s_′^DOWN^ = μ_s0_′). The absolute value of the absorption coefficient in the bottom layer was then calculated as μ_a_^DOWN^ = μ_a0_ + Δμ_a_^DOWN^.

Finally, from μ_a_^UP^ and μ_a_^DOWN^ at the two wavelengths, [O_2_Hb] and [HHb] in both layers were calculated by the Beer’s law using the extinction coefficients derived from the measurement of adult sheep^47^ for [HHb]: 1.672 cm^−1^ mM^−1^ (751 nm), 0.824 cm^−1^ mM^−1^ (839 nm), and for [O_2_Hb]: 0.752 cm^−1^ mM^−1^ (751 nm), 1.084 cm^−1^ mM^−1^ (839 nm).

## Conclusion

Our goal in this study was to improve fNIRS measurement accuracy and reliability in sheep to better understand the potential to noninvasively assess the cerebral activity of freely moving animals under different environmental conditions. Our findings confirmed that multi-distance CW-fNIRS allowed to noninvasively measure cerebral cortex activity in freely moving sheep and that the use of short and long-distance pairs of source-detector, coupled to a two-layer model for photon diffusion, can effectively discriminate extra-cerebral signals from cortical signals. Overall, these results indicate that with the current setup and probe placement we are primarily measuring the motor area of the sheep brain. Further investigations are needed to clarify whether fNIRS technique can be reliably applied to measure the deeply located cortical areas involved in processing affective state reactions. Future work must also consider possible factors that affect the accurateness of measurement such as probe location, and the species-specific neuroanatomical location corresponding to cognitive function.

## Supplementary information

Supplementary information.
