# Supplementary material for: Reliability of fNIRS for noninvasive monitoring of brain function and emotion in sheep
Source: Sci Rep. 2020 Sep 7;10:14726. doi: 10.1038/s41598-020-71704-5 (PMC7477174; doi:10.1038/s41598-020-71704-5)
Supplement: Supplementary file 1 — Supplementary information. [file 41598_2020_71704_MOESM1_ESM.pdf]

Supplementary data

Title:

Reliability of fNIRS for noninvasive monitoring of brain function and emotion in sheep

Authors:

Matteo Chincarini[1*], Emanuela Dalla Costa[2*], Lina Qiu[3,4*], Lorenzo Spinelli[5], Simona Cannas[2], Clara Palestrini[2], Elisabetta Canali[2], Michela Minero[2], Bruno Cozzi[6], Nicola Ferri[7], Daniele Ancora[3], Francesco De Pasquale[1], Giorgio Vignola[1], and Alessandro Torricelli[3,5]

Affiliations:

1 Università degli Studi di Teramo, Facoltà di Medicina Veterinaria, Teramo (Italy)

2 Università degli Studi di Milano, Dipartimento di Medicina Veterinaria, Milan (Italy)

3 Politecnico di Milano, Dipartimento di Fisica, Milan (Italy)

4 School of Software, South China Normal University, Guangzhou (China)

5 Istituto di Fotonica e Nanotecnologie, Consiglio Nazionale delle Ricerche, Milan (Italy)

6 Università degli Studi di Padova, Dipartimento di Biomedicina Comparata e Alimentazione, Padova (Italy)

7 Istituto Zooprofilattico Sperimentale dell'Abruzzo e del Molise G. Caporale, Teramo (Italy)

Corresponding authors:

mchincarini@unite.it, emanuela.dallacosta@unimi.it, alessandro.torricelli@polimi.it

* Equally contributed to the manuscript

Supplementary Section S1 Neuro-anatomical imaging of the sheep's head

We guided the positioning of the fNIRS probe by MRI data of the very same animals, and checked the functional origin of the signal by diffusion tensor imaging (DTI) MRI on the animal brain.

Preliminary MR scans were used to obtain relative position of the brain within the surrounding structures of the head. The MR data were acquired by means of an ESAOTE VETSCAN MR-Grande operating at 0.25 T. T1 weighted images were obtained with 2D Spin Echo T1 sequences with the following parameters: Slice Thickness = 3 mm, Repetition Time TR = 980 ms, EchoTime TE = 18 ms, Number of Averages: 2, Field of View FOV = 230x230 mm^2. A total of 27 slices were acquired. T2 weighted images were acquired through a 2D Fast Spin Echo T2 sequence with the following parameters: Slice Thickness: 3 mm, Repetition Time TR = 6030 mm, Echo Time TE = 100 ms, Number of Averages: 2, Field of View = 230x230 mm^2. A total of 27 slices were acquired.

For DTI we utilized the brains of six adult sheep, whose heads were collected at a commercial abattoir during routine slaughtering procedures. Slaughtering was performed according to the European Community Council directive (86/609/EEC) that regulates animal welfare during the whole commercial process and guarantees that animals are treated humanely and constantly monitored under mandatory official veterinary medical care. Once removed from the head, the brains were immediately fixed by immersion in cold buffered formalin. The time interval between death and removal of the brain varied between 10 and 20 minutes. The fixed brains were subsequently transported to the Department of Computer Science of the University of Verona for MR scans, using a 4.7 Tesla (T) magnet. Images were acquired with an Echo Planar Imaging (EPI) sequence with the following parameters: TR 20000 ms, TE 24.7 ms, FOV = 6.0x5.0 cm^2; MTX 120x100; isotropic in-plane resolution of 0.500 mm; slice thickness 1.0 mm; number of slice 80; EPI factor 11; NEX 6; 30 non-collinear directions acquired with a b-value of 3000 s/mm^2 and 5 b0 images for a total acquisition time of about 12 h 50 min (for further details and discussion of motor projections see Ref.[42]).

From MRI and DTI data we confirmed that the location of the fNIRS probe was over the motor area of the cortex (see Supplementary Figure SF1).

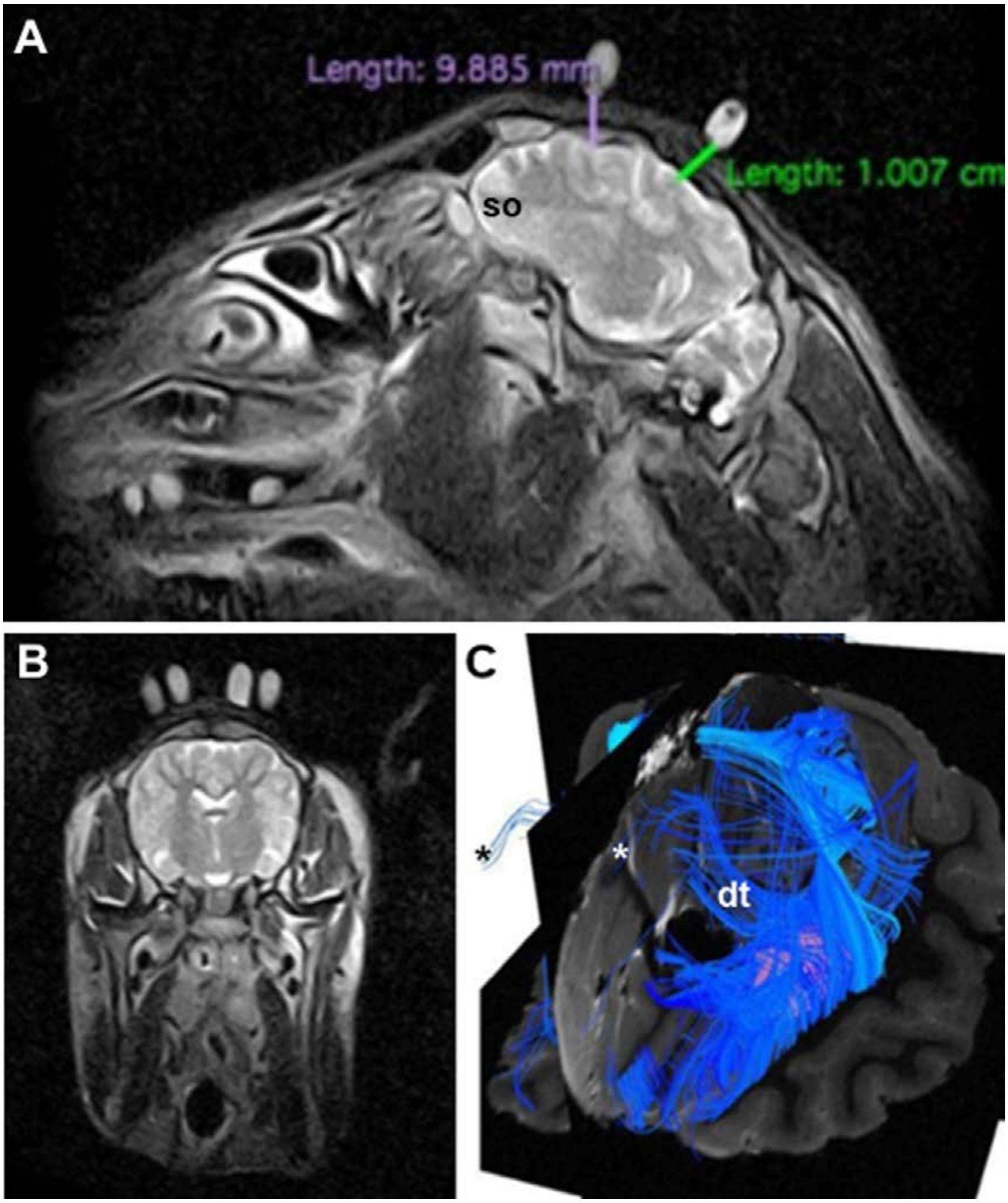

Supplementary Figure SF1. Images from MR scans (panel A, sagittal view; panel B, coronal view) and DTI (panel C). (so: supraorbital gyrus; dt: descending motor tracts; *: location of motor cortex, Brodmann area 4).

Supplementary Section S2 Baseline optical properties and DPF of the sheep's head

A time domain diffuse spectroscopy system was used to estimate the baseline optical properties (absorption coefficient, μ_{a0} and reduced scattering coefficient, μ'_{s0}) and the DPF of the head of the sheep. A supercontinuum fiber laser (SC450-6W, Fianium, UK) and a set of interference filters (Hard Coated OD 4 10nm Bandpass Filters, Edmund Optics Ltd. UK) were used to sequentially produce laser pulses (duration <100 ps, repetition rate 37 MHz, average power < 2 mW) at 671, 730, 780, and 830 nm. Multimode graded index glass (core diameter 0.1 mm) and plastic (core diameter 1.0 mm) optical fibers were used to inject and collect light into the head of the sheep, respectively. A home-made solid-state large area SiPM detector (developed at the Department of Physics of Politecnico di Milano)[48] and a time-correlated single photon counting board (SPC130 Becker-Hickl GmbH, Germany) were used to acquire the photon distribution of time of flights (DTOF). Measurements were performed with a source detector distance ρ = 25 mm over the right and left hemisphere of nine sheep (2337, 2339, 2345, 2349, 3908, 3909, 3911, 3913, 3919) while they were standing still, gently blocked by two people. The acquisition time was 1 s for each wavelength, while <10 s were needed to change wavelength and optimize the collected signal. Three repetitions for each position were acquired, resulting overall in about 4 minutes for each sheep.

A physical model for time-resolved reflectance in a homogeneous medium[45] was used to fit the DTOF after convolution with the instrument response function[49]. The fitting range was fixed at 90% and 1% of the peak on the leading and trailing edge of the DTOF. Values for μ_{a0} and μ_{s0}' were derived by averaging the results of the three repetitions in each hemisphere. From the DTOF we estimated the DPF as $\mathrm{DPF} = v < t >/\rho)$, where $v = c/n$ is the speed of light, $n = 1.4$ is the refractive index, and <t> is the photon mean time-of-flight (i.e. the barycenter or first order moment of the DTOF)[50–52].

Supplementary Figure SF2 shows the average and standard deviation values for the baseline optical properties (panel a and b) and for the DPF (panel c) at 671, 730, 780, and 830 nm as obtained by the time domain diffuse spectroscopy data. The difference between left and right hemisphere is not significant, therefore for data analysis and for simulations we have averaged the data over all sheep and channels. To obtain the μ_{s0}' background values at 751 nm and 839 nm (the wavelengths used by the used CW fNIRS device) we have fitted the μ_{s0}' data at 671 nm, 730 nm, 780 nm and 830 nm with an empirical approximation to the Mie theory, $\mu_{s0}' = a(\lambda/\lambda_0)^{-b}$ where *a* and *b* are parameters related to density and size of scatterers and λ is the

wavelength[53]. Similarly, we have fitted the μ_{a0} data at 671 nm, 730 nm, 780 nm and 830 nm to a linear combination of the contribution from HHb and O2Hb by means of the Beer's law (using the specific absorption of hemoglobin in sheep[47] and assuming a contribution from water 70%): average value for the absorption μ_{a0} are 0.178 cm^{-1} at 751 nm and 0.176 cm^{-1} at 839 nm, while the average values for the reduced scattering μ'_{s0} are 11.9 cm^{-1} at 751 nm and 10.4 cm^{-1} at 839 nm, as reported in in Supplementary Table ST1. We have also derived the values for the DPF at 739 nm and 851 nm by fitting the data at 671, 730, 780, and 830 nm to a quadratic polynomial function obtaining 6.6 and 6.0 at 751 nm and 839 nm, respectively.

Supplementary Table ST1. Average values and standard deviations of the head of the sheep for absorption coefficient, reduced scattering coefficient, and DPF at 671 nm, 730 nm, 780 nm, and 830 nm. Data at 739 nm and 851 nm are also shown as obtained from fitting of the μ_{s0}' data with an empirical approximation to the Mie theory[53], fitting the μ_{a0} data to Beer's law (using the specific absorption of hemoglobin in sheep[47], and fitting of DPF to a quadratic polynomial function.

	**671 nm**	**730 nm**	**780 nm**	**830 nm**	**751 nm**	**839 nm**
**μ_{a0} (cm^{-1})**	0.244±0.029	0.167±0.018	0.168±0.017	0.174±0.019	0.178±0.018	0.176±0.019
**μ_{s0}' (cm^{-1})**	13.8±1.4	12.3±1.2	11.4±1.1	10.6±1.1	11.9±1.2	10.4±1.1
**DPF (-)**	6.0±0.4	6.7±0.3	6.5±0.3	6.1±0.3	6.6±0.4	6.0±0.3

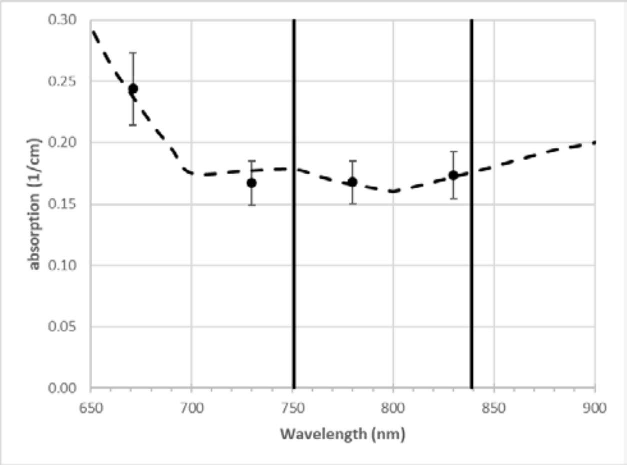

a)

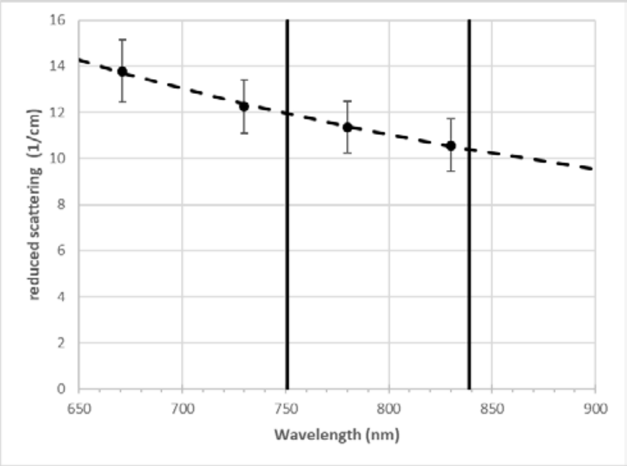

b)

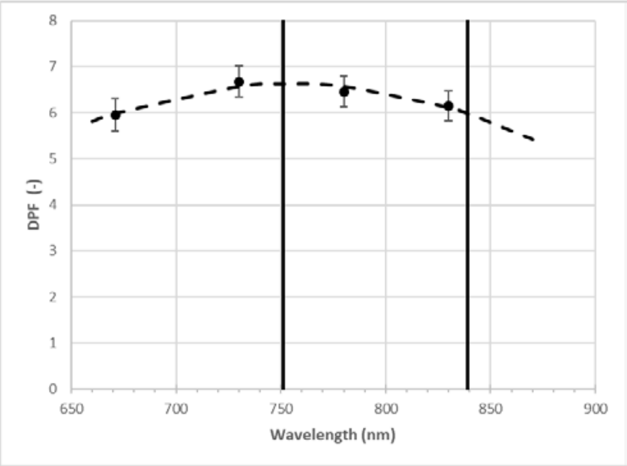

c)

Supplementary Figure SF2. Average values and standard deviations of the head of the sheep for absorption coefficient (a), reduced scattering coefficient (b), and DPF (c) at 671 nm, 730 nm, 780 nm, and 830 nm. Data are averaged over three repetitions in the left and right hemisphere. Dashed lines represent fitting of absorption coefficient with Beer's law (a), fitting of reduced scattering coefficient with empirical approximation to Mie theory (b), fitting of DPF to a quadratic polynomial function (c). Vertical lines represent the wavelengths (751 nm and 839 nm) used by the CW fNIRS device.

Supplementary Section S3 Depth sensitivity

In fNIRS studies, task related and task unrelated physiological changes occurring in the scalp can introduce confounding signals often leading to false positives and artefacts[54]. The adoption of a multi-distance approach is therefore essential in CW fNIRS to distinguish signals from shallow (e.g. scalp, skull and CSF) and deep (e.g. gray matter and white matter) layers in the head. This approach is justified by the physics of photon migration in diffusive media: in steady state (i.e. CW) the longer the source detector distance, the deeper is the average penetration depth[55]. Given an average value of scalp to cortex distance of about 15 mm[56] in fNIRS studies on adult human subjects, channels with relatively short source detector distance (e.g. $\rho \leq 10$ mm) are typically only able to reach surface layers, while channels with relatively long separation (e.g. $\rho \geq 30$ mm) can typically reach deeper layers in the head such as gray matter[57].

It has been reported that the cerebral cortex of sheep is located about 5 to 9 mm below the scalp[25]. According to anatomical and MRI measurements on the heads of several sheep of the same age (see Supplementary Section S1), for this study we could assume that the scalp and skull thickness of the sheep is on average 10 mm under the fNIRS probe. Therefore, in this study we used $\rho = 10$ mm and $\rho = 30$ mm as short and long channels, respectively. A shorter short distance (e.g. $\rho = 5$ mm) could not be used due to constraints in the shape of light emitter and detector of the CW fNIRS device we used. The use of a distance longer than 30 mm was prevented by the available space on the sheep head.

In order to have a rough estimate of the penetration depth of CW NIRS in our study, we calculated the mean maximum penetration depth ($\langle Z_{max}|\rho \rangle$) of photons for a homogenous slab in CW domain based on the diffusion equation (see Eq. (24) in Ref.[55]).

For a 30 mm thick homogenous slab with $\mu_a = 0.178$ cm^{-1} and $\mu_s' = 11.9$ cm^{-1}, the mean maximum depths are $\langle Z_{max}| \rho = 10 \text{ mm}\rangle \approx 5.48$ mm and $\langle Z_{max}| \rho = 30 \text{ mm}\rangle \approx 11.60$ mm. While for the same slab with $\mu_a = 0.176$ cm^{-1} and $\mu_s' = 10.4$ cm^{-1}, mean maximum depths are $\langle Z_{max}| \rho = 10 \text{ mm}\rangle \approx 5.53$ mm and $\langle Z_{max}| \rho = 30 \text{ mm}\rangle \approx 11.85$ mm.

To provide a representation of photon path in a diffusive two-layer medium we calculated the sensitivity maps for $\rho = 10$ mm and $\rho = 30$ mm by means of a perturbation model[58]. Assuming that the thickness of the upper (extra-cerebral) layer is 10 mm, we can clearly see from Supplementary Figure SF3 (left column) that the photons detected at $\rho = 10$ mm propagate mainly in the extra-cerebral layer (above the dotted line in Supplementary Figure SF3), while

photons detected at ρ = 30 mm can pass through the extra-cerebral layer and reach the bottom (cerebral) layer (below the dotted line in Supplementary Figure SF1).

Finally, 3D Monte Carlo simulations of photon migration in the sheep head were run in a realistic mesh taken from the same animal to validate the depth sensitivity (e.g. maximum penetration depth) of the adopted configuration. The mesh was created segmenting data from Spin-Echo T1 and Fast Spin-Echo T2 (FSE) MRI neurocranium scans of the sample PECORA_978_MONTANA. The voxel resolution of each scan was 0.4688 mm. T1 data was used to segment the outer structure of the head (better contrast tissue/air) while T2 was used to segment the brain (sharper signal from the white matter). The segmentation was accomplished extrapolating binary masks (head and brain) with Fiji[59] and fed a meshing routine implemented via iso2mesh[60]. The final mesh has 780,952 tetrahedral elements connecting 163,362 nodes. We took advantage of one of the most recent and accurate Monte Carlo simulation tools for the photon propagation, the Mesh-based Monte Carlo (MMC)[61], to resolve the photon diffusion within the realistic sheep head mesh. Two simulations with source-detector distances of 10 mm and 30 mm were performed in the frontal lobe of the sheep head. All the MC simulations were performed on a computer running Windows 10 (64 bit) with an Intel Xeon E5-2670 (2.6G) 8-core processor and the memory of 64 GB. The simulation time was about 1.4 h with MMC method for 4×10^8 photons in each simulation.

From the above results, it can be concluded that the short optical channel (ρ = 10 mm) used in our study can only detect hemodynamic information from the extra-cerebral tissue of the sheep head, while the long optical channel (ρ = 30 mm) can detect hemodynamic information from both the extra-cerebral tissue and the cerebral tissue.

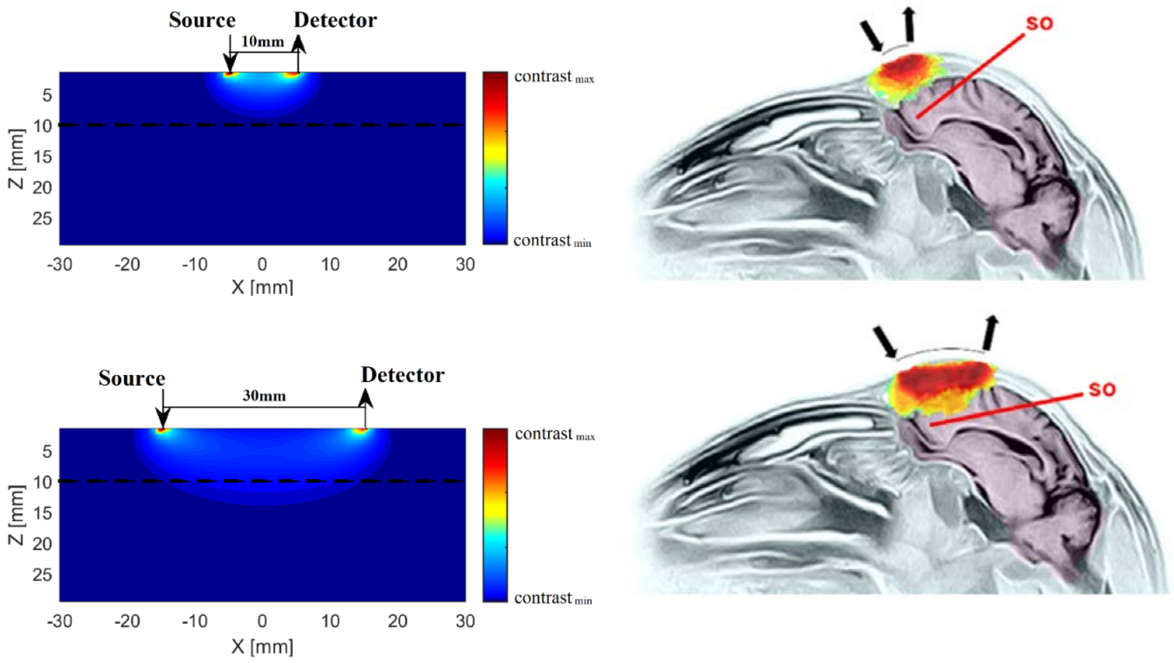

Supplementary Figure SF3. The CW sensitivity maps in a homogeneous slab (left column) and in a 3D realistic mesh of the sheep head (right column) detected at ρ =10 mm (top row) and ρ = 30 mm (bottom row). The right images overlay the anatomical segmentations (light blue for the head and light red for the brain) on top of the corresponding T2-MRI scan (inverse gray map for contrast enhancement). The position of the supraorbital gyrus (SO) is also reported. The sensitivity maps show clearly deeper brain penetration for photons detected at ρ = 30 mm.

Supplementary Section S4 Validation of the two step fitting method

Very often fNIRS signals of cortical activity are contaminated by surface (e.g. scalp and skull) interferences, which may lead to confounding results and potentially to fake activations and artifacts. A common solution to eliminate these artifacts is to directly measure the extra-cerebral signal through a short separation channel and remove it from the signal originating from the cortical region by means of statistical and regression methods like general linear model or principal component analysis, or also wavelet decomposition[62–66].

A limitation of this approaches is the fact that usually the short and long separation signals are both obtained by means of the modified Beer-Lambert law under the assumption that photons travel in a homogeneous volume[66,67].

We wanted to tackle the problem of discriminating superficial and cortical signal from a more physical point of view, by means of a multi-distance approach based on solution of the Diffusion Equation for photon migration in a two-layer geometry. This is similar to what already proposed with Monte Carlo instead of Diffusion Equation[68], and more generally to diffuse optical tomography approaches[69].

The fitting method adopted in this study is described in details in Section Materials and methods.

To verify the reliability of the proposed fitting method, we performed numerical simulations. Three different two-layer patterns of hemodynamic changes were generated using a physiologically relevant range of [O_2Hb] and [HHb] to mimic real changes in extra-cerebral and cerebral hemodynamics in sheep head during the fNIRS measurement. The steps of the forward modelling used to simulate hemodynamic changes are hereafter listed:

- The hemodynamic changes $\Delta[O_2Hb]^{up}(T)$ and $\Delta[HHb]^{up}(T)$ in up and down layer during stimulation period (considering a 10 s baseline period followed by 20 s stimulation, and 10 s recovery) were calculated[70] as a function of the experiment time T, and then converted into changes of the absorption coefficients in up and down layer $\Delta\mu_a^{up}(\lambda,T)$, $\Delta\mu_a^{down}(\lambda,T)$ using the Beer law[47].
- The absorption coefficients for the two layers were obtained by adding to $\Delta\mu_a^{up}(\lambda,T)$ and $\Delta\mu_a^{down}(\lambda,T)$ the baseline absorption coefficients $\mu_{a0}(\lambda)$, as obtained in Supplementary Section S2.
- The diffuse reflectance time courses at short $R_{short}(\lambda,T)$ and long $R_{long}(\lambda,T)$ source detector distance (ρ_{short} = 10 mm, ρ_{long} = 30 mm) were calculated using the two-layer solution of the photon Diffusion Equation, assuming the thickness of up layer s = 10

mm, and assuming that the scattering coefficient was constant and equal in both layers ($\mu_s'^{up}(\lambda,T) = \mu_s'^{down}(\lambda,T) = \mu_{s0}'(\lambda)$).

- Finally, the time courses for the changes in optical density at short and long source detector distance were calculated from $R_{short}(\lambda,T)$ and $R_{long}(\lambda,T)$:

  $\Delta OD_{short}(\lambda,T) = \log_{10}[R_{short}(\lambda,T_0)/R_{short}(\lambda,T)]$

  $\Delta OD_{long}(\lambda,T) = \log_{10}[R_{long}(\lambda,T_0)/R_{long}(\lambda,T)]$

The simulated time courses for [ΔO_2Hb] and [ΔHHb] in the up and down layer for the three different response patterns are shown in Supplementary Figure SF4: a) only the bottom layer (solid line) has hemodynamic changes, while the upper layer (dashed line) has only noise (Supplementary Figure SF4a); b) only the upper layer has hemodynamic changes, while the bottom layer has only noise (Supplementary Figure SF4b); c) both layers have similar hemodynamic changes, but the bottom response is relatively stronger (Supplementary Figure SF4c).

The fitting method described in Section Materials and Methods was then used to estimate [ΔO_2Hb](T) and [ΔHHb](T) from the simulated $\Delta OD(\lambda,T)$, and results are shown in right column of Supplementary Figure SF4.

By comparing the simulated hemodynamic changes and the corresponding estimated hemodynamic changes, we can conclude that the used fitting method can effectively separate signals coming from the upper (extra-cerebral) layer and the bottom (cerebral) layer. The maximum error between the simulated and estimated [ΔO_2Hb] and [ΔHHb] for the three different response patterns is <0.2 μM for the bottom layer and <0.03 μM for the up layer. These small errors may be due to the use of Beer Lambert's law when [ΔO_2Hb] and [ΔHHb] were converted to ΔOD, which is valid on the assumption that the head is a homogeneous medium.

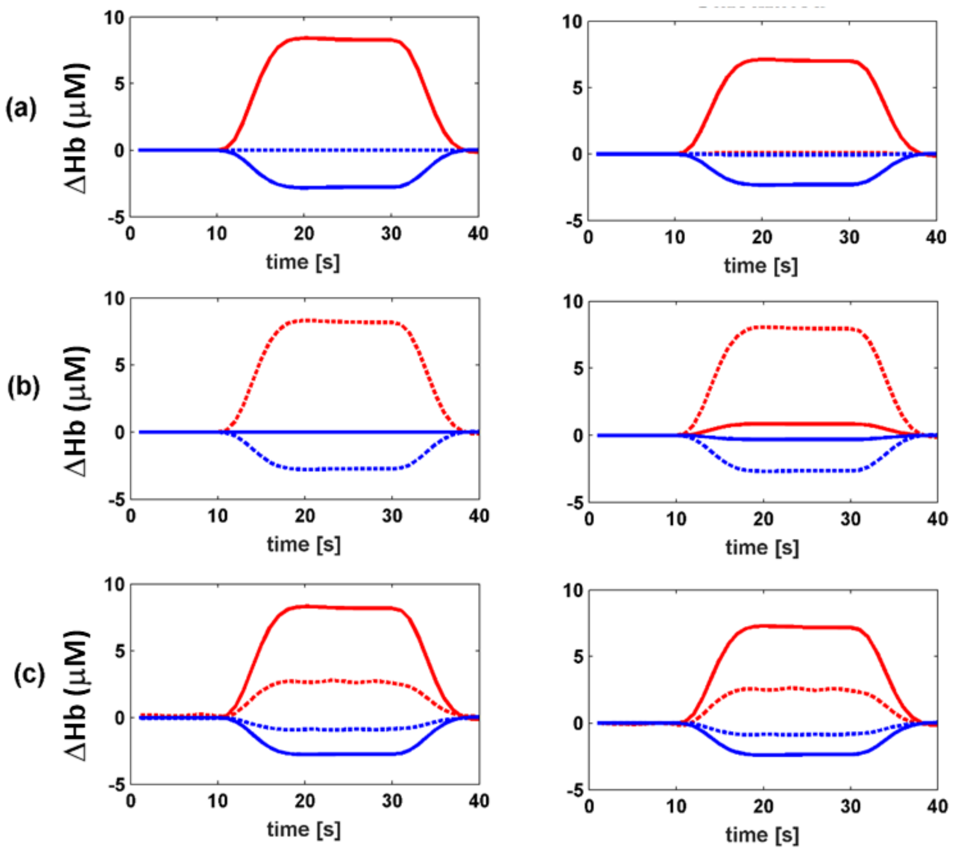

Supplementary Figure SF4. The simulated (left column) and estimated (right column) [ΔO_2Hb] (in red) and [ΔHHb] (in blue), in μM, for three different response patterns: (a) only the bottom layer (solid lines) has hemodynamic changes, while the upper layer (dashed lines) has only noise; (b) only the upper layer has hemodynamic changes and the bottom layer has only noise; (c) both layers have similar hemodynamic changes, but the bottom response is relatively larger.

Supplementary Section S5 Ethogram of sheep behavior

Supplementary Table ST2. Ethogram of sheep behavior.

Behavior	Description
Running	The sheep trots/gallops
Jumping	The sheep suddenly springs off the ground
Head shaking	The sheep shakes its head suddenly, violently and frequently either up/down or left/right
Chewing	The sheep works in the mouth with the teeth
Freezing	The sheep becomes rigid or motionless (standing still without moving) after the umbrella opening
Flight response	The sheep runs away after the umbrella opening

Supplementary Section S6 Block-average of hemoglobin changes in each sheep in the walking experiment

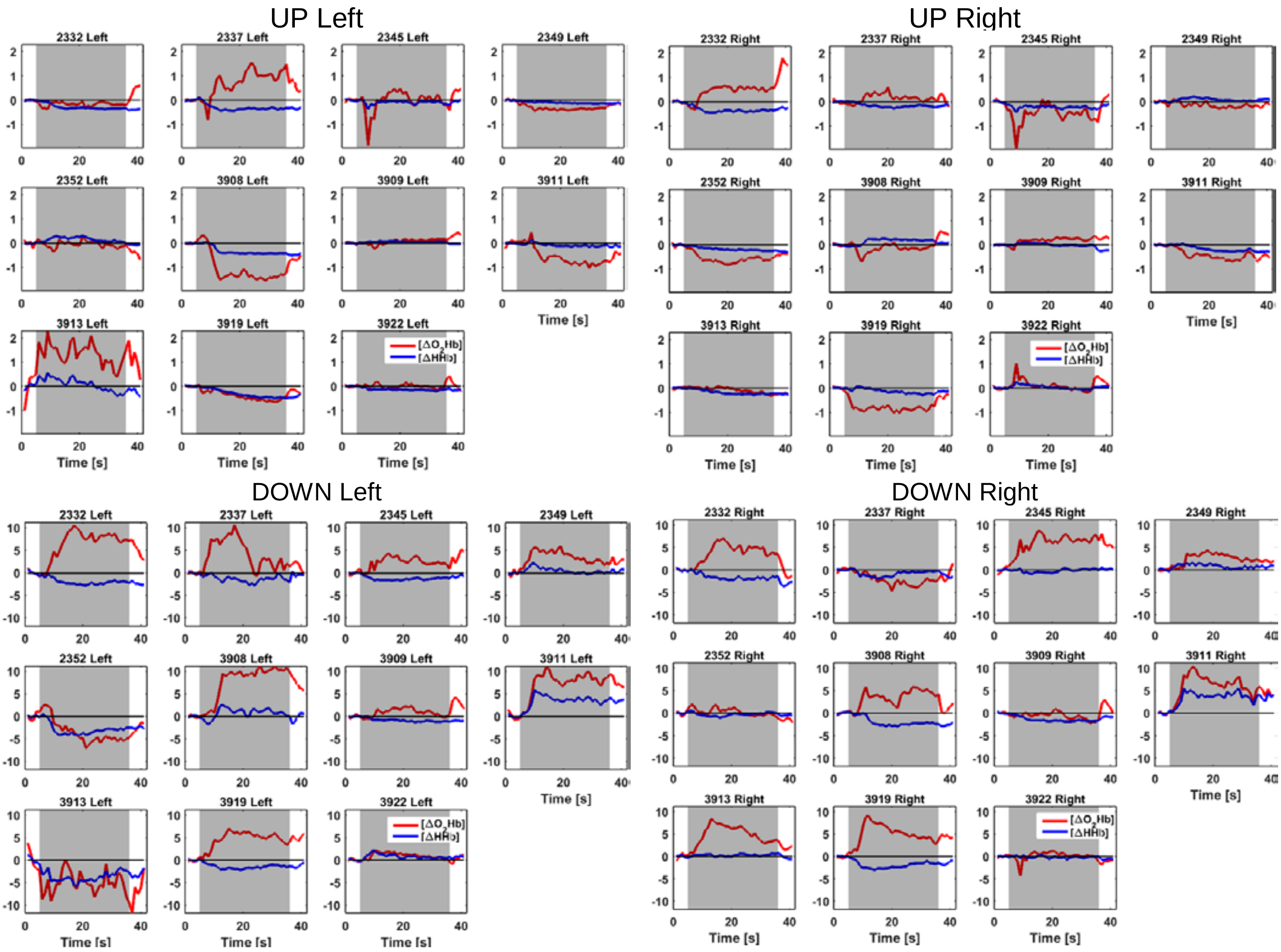

Supplementary Figure SF5. Block-average of $[\Delta O_2Hb]$ (red lines) and $[\Delta HHb]$ (blue lines), in µM, during the motor task for extra-cerebral (Up) tissue (top row) and cerebral (Down) tissue (bottom row) and for the left (left column) and right (right column) hemisphere in each sheep (sheep ID number on top of each panel). In every plot, the first 5 s are the baseline (sheep stand still), followed by 30 s walking (marked as gray area), and finally 5 s recovery period (sheep stand still). The horizontal black line in every sub-figure indicates the zero value.

Supplementary Section S7 Additional statistical analysis

Supplementary Table ST3. p-values of paired sample Student T-test comparing the hemodynamic response (i.e., oxygenated hemoglobin change [ΔO2Hb] and deoxygenated hemoglobin change [ΔHHb]) in the baseline (0 - 5 s) and in the period (10 - 25 s) for all experiments. The symbol '-' indicates that the p-value is not statistically significant ($p \geq 0.05$).

	**UP**				**DOWN**			
	**left**		**right**		**left**		**right**	
	**ΔO_2Hb**	**ΔHHb**	**ΔO_2Hb**	**ΔHHb**	**ΔO_2Hb**	**ΔHHb**	**ΔO_2Hb**	**ΔHHb**
**Motor task**	-	1.14E-08	2.05E-07	6.98E-08	1.22E-10	2.73E-09	1.92E-13	4.80E-09
**Startling test (all sheep)**	2.66E-08	-	1.04E-06	1.06E-03	1.39E-08	3.16E-04	1.92E-04	-
**Startling test (Move group)**	1.44E-05	-	6.06E-07	3.04E-03	2.44E-09	4.87E-05	4.35E-05	-
**Startling test (Stand group)**	-	8.38E-04	1.15E-02	7.29E-05	2.74E-02	1.16E-02	-	7.92E-08
